# A RALF22-like Peptide Coordinates Salt Tolerance and Disease Susceptibility in Poplar (*Populus davidiana* × *P. bolleana* ‘Shanxin’)

**DOI:** 10.3390/plants15101419

**Published:** 2026-05-07

**Authors:** Siying Pan, Xiaodie Huo, Ling Wu, Lichi Zhong, Qiang Cheng

**Affiliations:** State Key Laboratory of Tree Genetics and Breeding, Co-Innovation Center for Sustainable Forestry in Southern China, Nanjing Forestry University, Nanjing 210037, China

**Keywords:** *Populus davidiana* × *P. bolleana* ‘Shanxin’, RALF peptide, FERONIA, salinity tolerance, reactive oxygen species (ROS), disease susceptibility

## Abstract

Rapid alkalinization factor (RALF) peptides are recognized as multifunctional regulators of plant stress responses, yet their roles in woody species remain poorly defined. Here, we identified a RALF22-like peptide from poplar ‘Shanxin’ (*Populus davidiana* × *P. bolleana*; PdbRALF22-like) and investigated its roles in salt tolerance and disease resistance. Synthetic PdbRALF22-like peptide elicited a rapid ROS burst in poplar leaf discs. In *Nicotiana benthamiana*, which was otherwise unresponsive to the peptide, transient expression of either of two poplar FERONIA-like receptor kinases (*PdbFER-like-1* and *PdbFER-like-2*) enabled peptide-triggered ROS production, consistent with receptor-matched responsiveness in a heterologous context. Using CRISPR/Cas9, we generated a *PdbRALF22-like* knockout line and assessed salt tolerance in vitro and soil-grown assays. Under salinity, the mutant showed sustained rooting at high NaCl concentrations and improved growth relative to wild type. After 0.2 M NaCl treatment, soil-grown mutant plants exhibited reduced wilting and leaf injury. Evans Blue, DAB, and NBT staining indicated reduced membrane damage and lower accumulation of hydrogen peroxide and superoxide in the mutant. Significantly, the same knockout line displayed increased susceptibility to infection by the poplar leaf spot fungus, with larger lesions and higher pathogen biomass, accompanied by reduced ROS output and lower induction of the defense marker gene PdbPR1. Collectively, PdbRALF22-like negatively regulates salt tolerance while contributing positively to disease resistance, and represents a regulatory node linking salinity tolerance and disease susceptibility in poplar ‘Shanxin’, with poplar FER-like receptors providing a plausible route for peptide-triggered ROS signaling. This work expands our understanding of RALF peptide signaling in woody plants.

## 1. Introduction

In natural environments, diverse abiotic and biotic stresses severely limit woody plant productivity. Notably, soil salinity affects over 800 million hectares globally, posing a major threat to afforestation and plantation forestry [1]. Physiologically, salt stress impairs tree growth by disrupting ionic and osmotic homeostasis and inducing oxidative cellular damage [2]. In plantation and restoration settings, *Populus* spp. are widely used and often face not only salt stress but also pathogen pressure; for example, leaf spot caused by fungi of the genus *Marssonina* can lead to severe defoliation and growth inhibition in poplars [3]. Because woody plants experience multiple stresses over long life cycles, understanding how abiotic and biotic stress signals are integrated is essential.

Among plant signaling molecules, rapid alkalinization factor (RALF) peptides constitute one of the best-characterized families of secreted peptide signals and are broadly conserved across land plants [4]. RALFs were originally identified by their ability to induce rapid apoplastic alkalinization and are typically produced as precursor proteins containing an RRXL processing motif, yielding a mature, cysteine-rich peptide stabilized by disulfide bonds [4,5]. While recent literature has increasingly highlighted the involvement of RALF peptides in plant immunity and stress signaling, it is crucial to recognize that their primary and historically defining role lies in the regulation of basal plant growth and cell expansion. Mechanistically, RALF peptides act as negative regulators of plant growth by inducing rapid apoplastic alkalinization. This is achieved primarily through the direct inhibition of plasma membrane H+-ATPase (proton pump) activity [6]. Furthermore, RALF perception triggers robust intracellular signaling cascades, prominently including the activation of mitogen-activated protein kinases (MAPKs). Consequently, the emerging roles of RALF peptides in mediating defense responses should be viewed as a secondary functional diversification built upon their fundamental capacity to modulate essential growth and cellular expansion mechanisms [7]. In *Arabidopsis thaliana*, dozens of RALF genes have been annotated, and genetic and biochemical studies implicate RALF signaling in diverse growth- and stress-related processes [8].

Within the RALF family, phylogenetic analyses group AtRALF1, AtRALF22, AtRALF23, and AtRALF33 into a closely related clade [4]. Despite their sequence similarity, members of this clade exhibit pronounced functional divergence. AtRALF1 was among the first functionally characterized RALFs and inhibits primary root cell elongation through FERONIA-dependent signaling, thereby restricting cell expansion [9]. AtRALF22 and AtRALF23 have been implicated in salt stress responses via the LRX–RALF–FER module; notably, genetic perturbation of this pathway and overexpression of RALF22/23 are associated with altered salt stress tolerance [10]. Recent work also links RALF22 to immune activation, showing that RALF22 can trigger defense-associated outputs in a FER-dependent manner [11]. By contrast, AtRALF23 suppresses pattern-triggered immunity by inhibiting ligand-induced assembly of the FLS2/EFR–BAK1 receptor complex via FERONIA, and its close relative AtRALF33 has also been reported to dampen elicitor-induced ROS production [12]. Together, the AtRALF1/22/23/33 clade provides a useful framework for understanding how conserved RALF peptides diversify to modulate abiotic stress adaptation and immune signaling, while also influencing growth-related processes.

FERONIA (FER), a widely conserved *Catharanthus roseus* receptor-like kinase 1-like (CrRLK1L) receptor, represents a central signaling hub for RALF peptides [13]. In *Arabidopsis*, FER directly perceives multiple RALF peptides and transmits signals regulating cell expansion [9]. Structural studies confirm ligand recognition by the FER ectodomain within heterotypic complexes involving GPI-anchored LRE/LLG proteins [14]. Crucially, FER acts as a sensor that links cell-surface signaling to various stress outputs, primarily modulating ROS homeostasis and immune responses [15,16].

Despite extensive advances in model plants, the functions of RALF-like peptides in woody species remain largely unknown. Genome-wide surveys indicate that *Populus* encodes a large repertoire of malectin/malectin-like domain-containing proteins, including multiple CrRLK1L/FER-like receptor kinase candidates [17]. Whether poplar RALF-like peptides are perceived by FER-like kinases, how they modulate ROS-associated signaling under salinity, and whether they influence both salt tolerance and disease outcomes therefore remain open questions.

In this study, we identified a RALF22-like peptide from poplar ‘Shanxin’ *(Populus davidiana* × *P. bolleana*) and examined its evolutionary relationship to the AtRALF1/22/23/33 clade. We tested whether PdbRALF22-like signaling requires *Populus* FERONIA-like receptors by transiently expressing *PdbFER-like-1/2* in *Nicotiana benthamiana* and monitoring peptide-induced ROS. Using a CRISPR/Cas9-generated knockout line, we show that loss of *PdbRALF22-like* enhances salt tolerance but compromises resistance to the poplar leaf spot pathogen. Together, our results establish PdbRALF22-like as a FER-linked signaling component that connects ROS-associated outputs with opposing outcomes in abiotic and biotic stress responses in a woody species.

## 2. Results

### 2.1. Identification of a PdbRALF22-like Gene and Functional Characterization of Its Peptide

**Figure 1:**
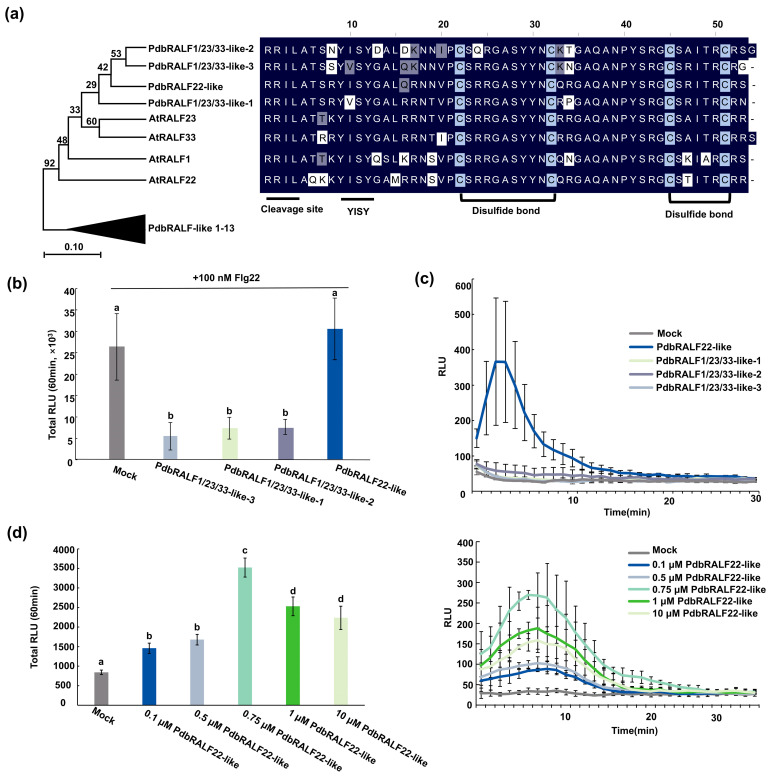
Identification and functional screening of a poplar RALF1/22/23/33-like subgroup and definition of PdbRALF22-like. (**a**) Maximum-likelihood phylogeny of AtRALF1/22/23/33 and predicted PdbRALF family members, together with an alignment of the C-terminal mature peptide region. The remaining PdbRALFs are collapsed (PdbRALF-like 1–13). Bootstrap support values (%) are indicated at nodes. (**b**) flg22-triggered ROS production in Pdb (‘Shanxin’) leaf discs co-treated with 100 nM flg22 and the indicated synthetic mature peptides (1 μM). Mock contained 100 nM flg22 and DMSO (1:1000, *v*/*v*) only. The y-axis represents total luminescence integrated over 60 min. Data are presented as mean ± SD from three independent experiments, each with six leaf discs per treatment. Different letters indicate statistically significant differences among treatments (one-way ANOVA followed by Tukey’s test, *p* < 0.05). Data are representative of three independent experiments with three biological replicates. (**c**) Kinetics of ROS production in Pdb leaf discs treated with the indicated synthetic mature peptides (1 μM) alone. Mock contained DMSO (1:1000, *v*/*v*) only. Curves show mean ± SD (*n* = 6 leaf discs). Data are representative of three independent experiments with three biological replicates. (**d**) Dose-dependent ROS production in Pdb leaf discs treated with the indicated concentrations (0.1, 0.5, 0.75, 1, and 10 μM) of synthetic PdbRALF22-like mature peptide. The bar graph represents total luminescence integrated over 60 min. Kinetics of ROS production are shown on the right. Mock contained DMSO (1:1000, *v*/*v*) only. Data are presented as mean ± SD (*n* = 6 leaf discs). Different letters indicate statistically significant differences among treatments (one-way ANOVA followed by Tukey’s test, *p* < 0.05). Data are representative of three independent experiments with three biological replicates.

Given that *A. thaliana* RALF22 has been implicated in salt-stress responses and can activate FER-dependent defense-associated outputs [10,11], we asked whether poplar encodes a closely related RALF22-like peptide. To identify RALF22-like candidates in *Populus davidiana* × *P. bolleana* (Pdb), we constructed a phylogenetic tree comprising AtRALF1, AtRALF22, AtRALF23, and AtRALF33 together with all predicted RALF family members from the Pdb genome (Table S1). Although no strict one-to-one ortholog of AtRALF22 was detected, four PdbRALFs clustered with AtRALF1/22/23/33 in a well-supported clade (bootstrap = 92%), defining a conserved RALF1/22/23/33-like subgroup shared between Arabidopsis and poplar. Alignment of the C-terminal mature peptide regions further showed that these four PdbRALFs share high sequence similarity with AtRALF1/22/23/33 (pairwise identities ≥ 72.2%) and retain hallmark RALF features, including the RRXL protease-processing motif and four conserved cysteine residues required for disulfide bond formation [4,5] (Figure 1a).

To assess the functional relevance of these candidate peptides, we synthesized the predicted mature peptides and tested their effects on ROS production in Pdb leaf discs. When co-applied with flg22, three peptides significantly reduced flg22-triggered ROS production (Figure 1b), whereas one peptide did not significantly alter the flg22-induced ROS burst. Importantly, this peptide alone elicited a robust and transient ROS burst, peaking approximately 3–5 min after treatment and returning to baseline thereafter (Figure 1c), whereas the other three peptides did not induce ROS above the mock control.

Based on these activity profiles, the peptide that independently induced ROS was designated PdbRALF22-like (NCBI accession: PX722030), and the remaining three peptides were named PdbRALF1/23/33-like-1, -2, and -3 (NCBI accession: PX722027, PX22028 and PX22029), respectively. This nomenclature reflects both their phylogenetic relationship to the AtRALF1/22/23/33 clade and their contrasting effects on flg22-triggered ROS in poplar, consistent with the FER-dependent immune-activating activity reported for RALF22 and the immune-suppressive activity reported for RALF23 in *Arabidopsis* [11,12].

To further characterize the elicitor activity of PdbRALF22-like, we evaluated its dose-dependence. The amplitude of the ROS burst increased in a concentration-dependent manner, reaching a maximum between 0.75 μM and 1 μM, confirming the specific and saturable nature of the peptide recognition (Figure 1d), which is consistent with the results in *Arabidopsis* [11].

### 2.2. PdbFER-like Receptors Confer PdbRALF22-like Responsiveness in N. benthamiana

**Figure 2:**
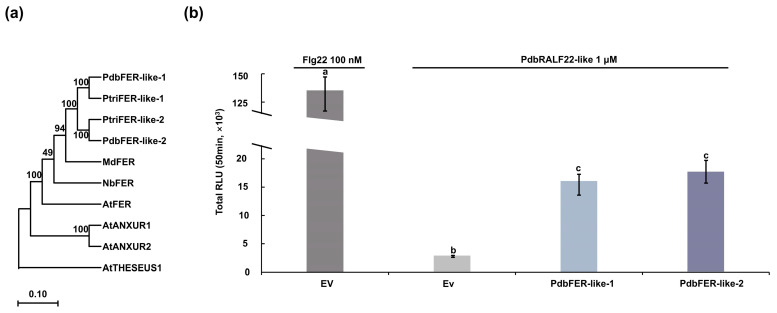
PdbFER-like receptors confer PdbRALF22-like responsiveness in *Nicotiana benthamiana*. (**a**) Maximum-likelihood phylogeny of FER/FER-like proteins from *Populus davidiana* × *P. bolleana* (Pdb) and *P. trichocarpa*, together with functionally characterized FER proteins from other species. The *A. thaliana* CrRLK1L members ANXUR1 (AtANXUR1), ANXUR2 (AtANXUR2), and THESEUS1 (THE1) were included as outgroups. Bootstrap support values (%) from 1000 replicates are indicated at nodes. Gaps and missing data of PdbFER family members were treated using the complete deletion option. (**b**) ROS production in *N. benthamiana* leaf discs transiently expressing *PdbFER-like* receptors. Leaf discs were prepared from leaves infiltrated with *Agrobacterium* carrying an empty vector (EV) or constructs expressing *PdbFER-like-1/2*, and ROS production was quantified as total luminescence integrated over 50 min after treatment with flg22 (100 nM) or PdbRALF22-like peptide (1 μM), as indicated. Data are presented as mean ± SD from three independent experiments, each with six leaf discs per treatment. Different letters indicate statistically significant differences among treatments (one-way ANOVA followed by Tukey’s test, *p* < 0.05). Note the y-axis break. The y-axis represents total Relative Light Units (RLU) integrated over 50 min. A y-axis break is utilized to clearly visualize the baseline responses of the EV and non-responsive treatments alongside the massive ROS burst triggered by the flg22 positive control. Data are representative of three independent experiments with three biological replicates.

To identify candidate receptors that could underlie the poplar response to PdbRALF22-like, we performed reciprocal BLAST (BioEdit, Version 7.0.9.0) searches using *A. thaliana* FERONIA (AtFER) as the query and retrieved two FER homologs from each of the *P. trichocarpa* and Pdb proteomes. The two Pdb homologs were designated PdbFER-like-1 (NCBI accession: PX722025) and PdbFER-like-2 (NCBI accession: PX722026). We then constructed a maximum-likelihood phylogeny including FER proteins from *A. thaliana*, apple, and *N. benthamiana*—species in which FER has been functionally characterized as a RALF receptor—together with the *Populus* FER homologs. The analysis placed the Pdb and *P. trichocarpa* FER proteins within the FER lineage with strong support (bootstrap = 100%) (Figure 2a). PdbFER-like-1 grouped with its *P. trichocarpa* counterpart, and PdbFER-like-2 grouped with the second *P. trichocarpa* homolog, forming two strongly supported sister pairs. The two Pdb FER homologs were also highly similar to each other (amino-acid identity ≥ 88.8%), consistent with retention of duplicated *FER-like* genes in *Populus*.

To test whether these poplar FER-like receptors are sufficient to confer responsiveness to PdbRALF22-like, we transiently expressed *PdbFER-like-1* or *PdbFER-like-2* in *N. benthamiana* leaves and subsequently challenged leaf discs with the synthetic PdbRALF22-like mature peptide. Expression of either poplar receptor enabled a robust ROS burst upon peptide treatment (Figure 2b), whereas leaf discs infiltrated with Agrobacterium carrying the empty vector remained unresponsive. No significant difference in ROS output was detected between PdbFER-like-1 and PdbFER-like-2 under these conditions. Together, these results show that heterologous expression of PdbFER-like receptors is sufficient to confer PdbRALF22-like responsiveness in *N. benthamiana*, and suggest functional redundancy between the two poplar FER-like receptors in this assay.

### 2.3. Generation and Identification of PdbRALF22-like Mutants

**Figure 3:**
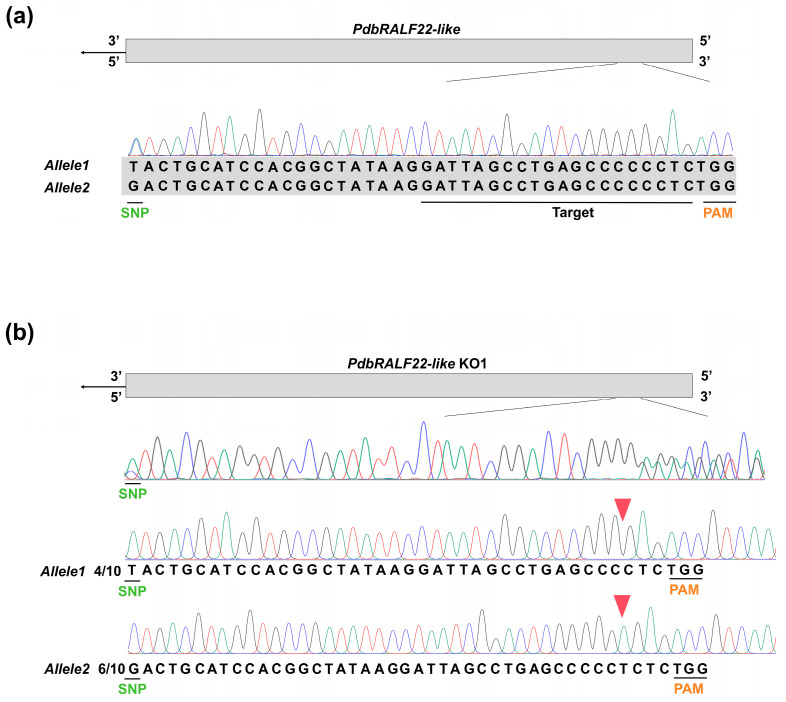
Generation and genotyping of a CRISPR/Cas9-edited *PdbRALF22-like* knockout line. (**a**) Schematic of the CRISPR/Cas9 target region within the PdbRALF22-like coding sequence. The protospacer, the adjacent PAM, and a nearby SNP marker are indicated. Colored lines represent the sequencing signal peaks of the four nucleotides (A, T, C, G). (**b**) Sanger sequencing chromatograms and TA-clone sequencing of the PCR amplicon spanning the target site in PdbRALF22-like KO1 (L3). Allele assignment was based on the SNP marker. Numbers indicate the proportion of sequenced TA clones supporting each edited allele. Colored lines represent the sequencing signal peaks of the four nucleotides (A, T, C, G). Red triangles indicate the position of CRISPR/Cas9-induced mutations in the target gene.

To investigate the biological function of PdbRALF22-like, we generated CRISPR/Cas9-mediated knockout mutants in Pdb. The CRISPR/Cas9 target region within the *PdbRALF22-like* coding sequence, including the protospacer, the adjacent PAM, and an allele-specific SNP marker, is shown in Figure 3a. Putative transformants were first confirmed by PCR using Cas9-specific primers (Figure S1). We then amplified a genomic fragment spanning the target site and the SNP marker, and performed Sanger sequencing to screen for edited lines. One line (L3) displayed clear editing signatures in the chromatograms, and subsequent TA cloning and sequencing of individual amplicons resolved a stable biallelic genotype: one allele carried a 2 bp deletion (ΔCC) located 2 bp upstream of the PAM, whereas the other allele harbored a single-nucleotide insertion (+T) located 3 bp upstream of the PAM (Figure 3b). Both mutations introduce frameshifts within the coding sequence, with the frameshift occurring after the 33rd codon in the region corresponding to the mature peptide. This line was therefore designated *PdbRALF22-like* KO1.

### 2.4. Knockout of PdbRALF22-like Enhances Salt Tolerance in Poplar

**Figure 4:**
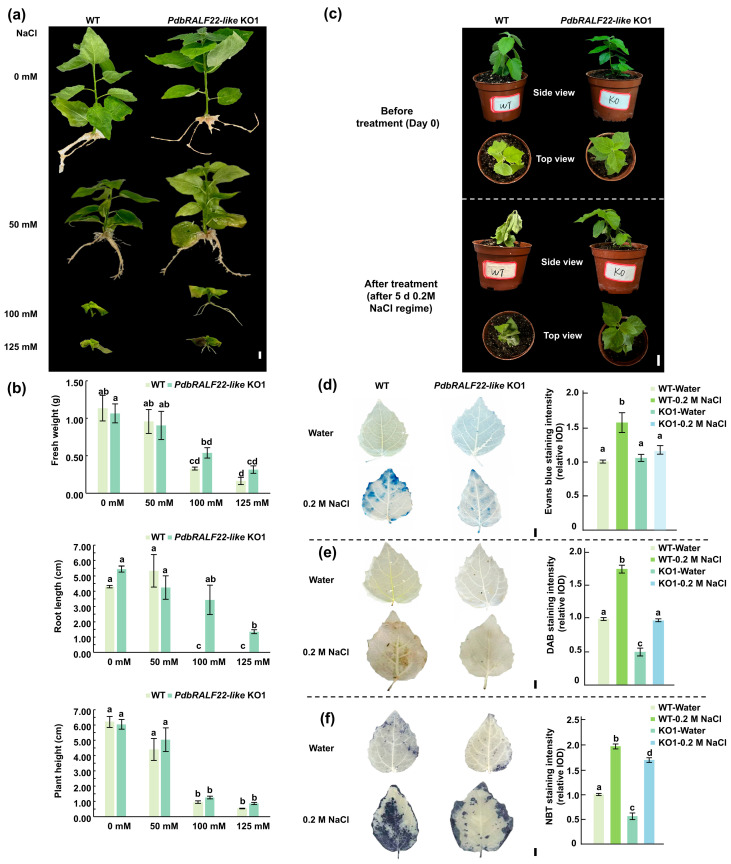
Knockout of PdbRALF22-like enhances salt tolerance in poplar. (**a**) Representative phenotypes of WT and PdbRALF22-like KO1 plants grown for 1 month on 1/2 MS rooting medium supplemented with 0, 50, 100, or 125 mM NaCl. (**b**) Quantification of fresh weight, root length, and plant height of WT and KO1 plants after 1 month of growth on 1/2 MS rooting medium under the indicated NaCl concentrations. Data are presented as mean ± SD (*n* = 3 biological replicates). Different letters indicate statistically significant differences among treatments (one-way ANOVA followed by Tukey’s test, *p* < 0.05). Data are representative of three independent experiments with three biological replicates. (**c**) Salt-stress phenotypes of soil-grown WT and KO1 plants treated with 0.2 M NaCl for five consecutive days. Phenotypes were documented on the day treatment ended. For each genotype and time point, plants are shown as a side view (upper images) and a top view (lower images). (**d**–**f**) Histochemical staining of fully expanded 3rd–5th leaves from WT and KO1 plants under control (water) or salt stress (0.2 M NaCl for 8 h). The bar graphs display the staining intensity (relative IOD) of the histochemical assays. Evans Blue staining (**d**) indicates membrane damage, DAB staining (**e**) detects H_2_O_2_ accumulation, and NBT staining (**f**) detects superoxide accumulation. Representative images are shown. Scale bars = 1 cm in (**a**,**c**–**f**).

To evaluate the contribution of PdbRALF22-like to salt tolerance, we compared the performance of the knockout line KO1 with wild-type (WT) plants under salinity. In an in vitro assay, apical shoot cuttings were transferred to 1/2 MS rooting medium supplemented with 0, 50, 100, or 125 mM NaCl for 1 month. Under non-saline conditions, KO1 and WT showed no obvious differences in morphology, fresh weight, root length, or shoot height. At 50 mM NaCl, both genotypes rooted and grew normally. By contrast, clear divergence was observed at 100 mM NaCl: WT plants largely failed to initiate roots, whereas KO1 plants maintained robust rooting (Figure 4a). Consistently, KO1 accumulated significantly higher fresh weight than WT at this concentration (1.63-fold) (Figure 4b). Even at 125 mM NaCl, KO1 plants still produced roots, although overall growth was reduced.

We next assessed salt tolerance under soil-grown conditions. Plants with comparable morphology were transferred to pots and grown for 30 days, then treated with 0.2 M NaCl for five consecutive days. Phenotypes were documented on the day the 5-day NaCl treatment ended. WT plants showed pronounced salt-stress symptoms, including wilting, leaf chlorosis and curling, and substantial leaf abscission, whereas KO1 plants displayed markedly milder symptoms, with limited leaf yellowing/curling and little to no leaf drop (Figure 4c).

To further evaluate salt-induced cellular damage and ROS accumulation, leaves from water- and salt-treated plants were subjected to Evans Blue, DAB, and NBT staining. Under control conditions, KO1 and WT leaves showed comparable staining patterns. Following salt stress, KO1 leaves consistently exhibited weaker staining intensity and smaller stained areas across all assays, indicating reduced membrane injury and lower accumulation of hydrogen peroxide and superoxide relative to WT (Figure 4d–f).

Collectively, these results demonstrate that loss of *PdbRALF22-like* enhances salt tolerance in poplar, as reflected by sustained rooting capacity under salinity, reduced visible injury in soil-grown plants, and attenuated salt-associated ROS accumulation and membrane damage.

### 2.5. Knockout of PdbRALF22-like Reduces Disease Resistance in Poplar

**Figure 5:**
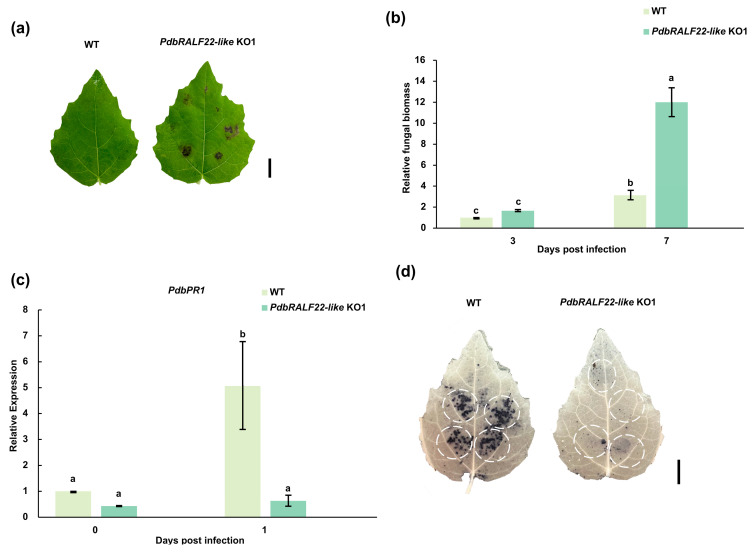
Knockout of *PdbRALF22-like* compromises resistance to the poplar leaf spot fungus. (**a**) Representative disease symptoms on detached leaves from WT and PdbRALF22-like KO1 plants at 7 days post inoculation (dpi) with *M. brunnea* (Mb). (**b**) Relative fungal biomass in WT and KO1 leaves at 3 and 7 dpi, quantified by qPCR as the abundance of *MbEF1-α* normalized to the poplar reference gene *PdbEF1-α*. (**c**) RT-qPCR analysis of *PdbPR1* expression in WT and KO1 leaves at 0 and 1 dpi. Transcript levels were normalized to *PdbEF1-α* and are presented relative to the WT 0 dpi sample. Different letters indicate statistically significant differences among treatments (one-way ANOVA followed by Tukey’s test, *p* < 0.05). (**d**) NBT staining showing superoxide accumulation at inoculation sites at 1 dpi; dashed circles indicate the inoculation sites. Bars indicate the mean ± standard error, SE (from three independent experiments). Data are representative of three independent experiments with three biological replicates. Scale bars = 1 cm in (**a**,**d**).

To assess whether PdbRALF22-like contributes to disease resistance, detached leaves from WT and KO1 plants were inoculated with the poplar leaf spot fungus, *M. brunnea*. Compared with WT, KO1 leaves developed larger necrotic lesions and more severe disease symptoms (Figure 5a), indicating increased susceptibility upon loss of *PdbRALF22-like*. We next quantified fungal biomass by qPCR. At an early stage of infection (3 days post inoculation, dpi), fungal biomass did not differ significantly between WT and KO1. However, by 7 dpi, KO1 leaves accumulated significantly higher fungal biomass than WT (3.84-fold) (Figure 5b), consistent with enhanced pathogen colonization and/or proliferation in the absence of PdbRALF22-like.

To examine early defense-associated outputs, we analyzed the expression of the defense marker gene *PdbPR1*. RT-qPCR showed that *PdbPR1* was strongly induced in WT at 1 dpi, whereas this induction was markedly reduced in KO1 (Figure 5c). In addition, NBT staining revealed stronger superoxide accumulation at infection sites in WT leaves, while KO1 leaves displayed a substantially weaker signal at 1 dpi (Figure 5d). Collectively, these results indicate that loss of *PdbRALF22-like* attenuates early ROS-associated defense responses and is associated with reduced resistance to *M. brunnea* infection in poplar.

## 3. Discussion

### 3.1. Divergence Within the Poplar RALF1/22/23/33-like Clade

In *A. thaliana*, AtRALF1, AtRALF22, AtRALF23, and AtRALF33 form a closely related group with high sequence similarity in their mature peptide regions and have been implicated in both growth-related processes and stress-associated signaling [4]. However, RALF genes undergo frequent duplication and functional diversification; thus, phylogenetic relatedness does not necessarily imply strict one-to-one functional orthology across species [18]. Consistent with this notion, our phylogenetic analysis did not resolve a clear one-to-one correspondence between *Arabidopsis* AtRALF1/22/23/33 and the four highly similar poplar RALFs that clustered within the same RALF1/22/23/33-like subgroup.

In *Arabidopsis*, members of this clade are nonetheless functionally partitioned in immune-associated ROS outputs: AtRALF23 suppresses PTI-associated signaling through FERONIA-dependent mechanisms [12], whereas RALF22 has been reported to trigger FER-dependent defense-associated outputs, including ROS [11]. Guided by this clade-level functional contrast, we performed functional screening in poplar using ROS as an early readout. Interestingly, the four poplar peptides also separated into two activity types: three peptides reduced flg22-triggered ROS, whereas PdbRALF22-like alone was sufficient to elicit a rapid and transient ROS burst in poplar leaf discs.

Together, these results do not support a strict one-to-one functional equivalence between specific *Arabidopsis* and poplar RALFs, but they do point to a conserved capacity within the RALF1/22/23/33-like subgroup to diversify into ROS-suppressing versus ROS-eliciting activities. Notably, this activity partitioning occurs among a set of highly similar mature peptides: across the eight members analyzed here (four from *Arabidopsis* and four from poplar), the mature peptide regions are strongly conserved. This combination—high sequence conservation yet divergent ROS outputs—suggests that RALF peptides may encode signaling “bias” through a limited number of key residues rather than broad sequence divergence.

### 3.2. Receptor-Matched Responsiveness and Duplicated FER-like Receptors

Our heterologous assays indicate that responsiveness to PdbRALF22-like is receptor-matched and species dependent. FERONIA (FER), a CrRLK1L receptor kinase, is a central component of RALF signaling in *A. thaliana* and can regulate immune-associated outputs [9,12,19]. In our experiments, exogenous PdbRALF22-like did not elicit detectable ROS in *N. benthamiana*, whereas transient expression of either *PdbFER-like-1* or *PdbFER-like-2* enabled robust peptide-triggered ROS. These data support receptor compatibility as a key determinant of cross-species responsiveness, and show that a cognate poplar receptor can confer signaling competence in a heterologous cellular context.

The two poplar receptors produced similar ROS outputs under the tested conditions, consistent with functional redundancy for this readout. Poplar genomes have experienced lineage-specific whole-genome duplication, and duplicated signaling genes are often retained [20]. The presence of two FER-like homologs in both *P. trichocarpa* and poplar ‘Shanxin’, and their grouping into two supported sister pairs, is consistent with retention of duplicated *FER-like* genes in poplar. Whether these paralogs differ in other outputs, expression patterns, or ligand specificities remains to be determined.

### 3.3. A Trade-Off Node Linking Salt Tolerance and Disease Resistance

Our genetic evidence in poplar ‘Shanxin’ shows that loss of *PdbRALF22-like* has opposing consequences for salinity tolerance and fungal disease resistance, with knockout of *PdbRALF22-like* enhancing salt tolerance in both tissue-culture and soil-grown assays, including sustained rooting at high NaCl concentrations and reduced visible injury after the 0.2 M NaCl regime. Consistently, histochemical staining indicated reduced ROS accumulation and attenuated membrane injury under salt stress. Conversely, loss of *PdbRALF22-like* reduced resistance to the poplar leaf spot fungus, with larger lesions and increased fungal biomass at later infection stages, accompanied by reduced *PdbPR1* induction and weaker ROS-associated readouts during infection. Together, these results reveal a trade-off between salinity tolerance and fungal disease resistance associated with loss of *PdbRALF22-like* in poplar ‘Shanxin’. While our genetic evidence positions PdbRALF22-like as a critical node coordinating salt tolerance and disease resistance, the mechanistic basis of this regulation requires further validation. The contrasting phenotypes could result from direct regulation, where PdbRALF22-like binding to its cognate FER-like receptors triggers a rapid, specific intracellular signaling cascade (such as ROS bursts and MAPK activation) that prioritizes defense pathways at the expense of abiotic stress tolerance. Alternatively, the regulation may be indirect. For instance, constitutive RALF signaling might alter cell wall integrity or alter the baseline apoplastic pH, indirectly predisposing the plant to susceptibility under salt stress while maintaining a primed state against fungal penetration. Future biochemical studies, including the identification of specific downstream phosphoproteins and target genes, will be essential to decipher the direct and indirect regulatory networks of this peptide.

In *A. thaliana*, AtRALF22 has been linked to salt-stress responses within the LRX–RALF22/23–FER framework [10], and overexpression of *RALF22* or *RALF23* increases salt sensitivity, supporting a negative role for elevated RALF22/23 signaling in salinity tolerance. By contrast, AtRALF22 has also been reported to promote FER-dependent defense-associated outputs [11], offering a plausible explanation for the positive immune contribution observed here, although the corresponding interaction partners and pathway context remain to be defined in poplar. Taken together, our results suggest that a shared RALF-centered framework may tune the balance between salinity tolerance and disease resistance in two evolutionarily distant plants with contrasting lifestyles, *Arabidopsis* and poplar trees.

## 4. Materials and Methods

### 4.1. Plant Materials and Growth Conditions

The hybrid poplar ‘Shanxin’ (*Populus davidiana* × *P. bolleana*) was used in this study. In vitro plantlets were maintained under sterile conditions on Murashige and Skoog (MS, Coolaber, Beijing, China) medium (pH 5.8) supplemented with 0.3 mg L^−1^ indole-3-acetic acid (IAA), 3% (*w*/*v*) sucrose, and 0.7% (*w*/*v*) agar. All chemical reagents, medium components, antibiotics and plant hormones used in this study were purchased from Coolaber (Beijing, China), unless otherwise specified. Cultures were grown in a growth chamber under a 16 h light/8 h dark photoperiod at 24 °C. For greenhouse experiments, four-week-old in vitro-grown poplar plantlets were acclimated and transplanted into soil, and grown under a 16 h light/8 h dark photoperiod at 24 °C. *N. benthamiana* plants were grown in the same greenhouse under the same conditions.

### 4.2. Identification of PdbRALFs and Peptide Synthesis

To identify members of the rapid alkalinization factor (RALF) gene family in the *P. davidiana* × *P. bolleana* (Pdb) genome (assembly accession GCA_027475595.1), annotated *A. thaliana* RALF protein sequences were retrieved from TAIR and used as query sequences. Iterative BLASTP searches were performed against the Pdb protein dataset (E-value ≤ 1 × 10^−10^). Candidate sequences obtained in the first round were used as new queries for subsequent rounds until no additional candidates were identified. All candidates were then annotated using the InterProScan (https://www.ebi.ac.uk/interpro/search/sequence/, accessed on 26 April 2026) web server, and only sequences containing the characteristic RALF domain (InterPro: IPR008801) were retained (Table S1).

Based on the predicted mature peptide regions, four PdbRALF peptides with high sequence similarity to AtRALF1/22/23/33 were chemically synthesized by GenScript Biotech Corporation (Nanjing, China) and purified to >90% purity (HPLC), and were used for subsequent experiments.

### 4.3. Reactive Oxygen Species Measurement

ROS production was quantified using a luminol-based assay [21]. Leaf discs (0.5 cm diameter) were punched from fully expanded leaves. For poplar, discs were collected from the 3rd–5th leaves (counting from the apex) of plants grown in soil for one month after transplantation. For *N. benthamiana*, discs were collected from leaves of four-week-old plants. For each sample, six leaf discs were placed in each well of a 96-well plate containing 200 μL sterile distilled water and incubated overnight. Before measurement, the water was replaced with 200 μL assay solution containing 100 μM luminol (Sigma-Aldrich, St. Louis, MO, USA), 20 μg mL^−1^ horseradish peroxidase (Sigma-Aldrich), and peptides at the indicated concentrations. Bioluminescence was recorded using a GloMax™ 96 Microplate Luminometer (Promega, Tokyo, Japan), and ROS production was expressed as relative light units (RLU).

### 4.4. Binary Vector Construction and Transient Expression in N. benthamiana

The coding sequences (CDSs) of *PdbFER-like-1* and *PdbFER-like-2* were amplified from cDNA prepared from wild-type poplar ‘Shanxin’ and cloned into the binary expression vector pH35GS under the control of the CaMV 35S promoter. All constructs were verified by Sanger sequencing. The resulting plasmids were introduced into *Agrobacterium tumefaciens* strain GV3101 by electroporation. Primers used for vector construction are listed in Table S2.

For transient expression, *Agrobacterium* cultures were grown overnight, collected by centrifugation, and resuspended in infiltration buffer containing 10 mM MES and 10 mM MgCl_2_ (pH 5.7), supplemented with 150 μM acetosyringone. Bacterial suspensions were adjusted to an OD_600_ of 0.4 and incubated at room temperature for 3 h prior to infiltration. Leaves of four-week-old *N. benthamiana* plants were infiltrated using a needleless syringe. Plants were maintained under normal growth conditions, and infiltrated tissues were used for subsequent assays at 2 days post infiltration unless otherwise indicated.

### 4.5. CRISPR/Cas9-Mediated Knockout of PdbRALF22-like

The CRISPR/Cas9 construct targeting PdbRALF22-like was generated using the pYLCRISPR system as described previously [22]. Briefly, target spacer sequences were cloned into the BsaI site of the sgRNA entry plasmid pYLsgRNA-AtU3d, and the resulting sgRNA cassette(s) were assembled into the BsaI site of the pYLCRISPR/Cas9 binary vector (Table S2).

Poplar ‘Shanxin’ was transformed using *A. tumefaciens* strain EHA105 carrying the pYLCRISPR/Cas9-PdbRALF22-like binary vector. Leaf segments (~0.5 cm × 0.5 cm) excised from four-week-old sterile plantlets were immersed in an Agrobacterium suspension (OD_600_ = 0.6), blotted dry, and co-cultivated for 2 days. Explants were then transferred to shoot induction medium (MS basal salts, pH 5.7, supplemented with 0.1 mg L^−1^ 1-naphthaleneacetic acid, 0.2 mg L^−1^ 6-benzyladenine, 0.01 mg L^−1^ thidiazuron, 50 mg L^−1^ kanamycin, and 400 mg L^−1^ timentin, with 7 g L^−1^ agar) for selection and elimination of *Agrobacterium*. Kanamycin-resistant shoots were excised and transferred to rooting medium (1/2 MS salts, pH 5.7, containing 0.3 mg L^−1^ indole-3-butyric acid, 20 mg L^−1^ kanamycin, and 400 mg L^−1^ timentin, with 0.7 g L^−1^ agar). Well-rooted, kanamycin-resistant plantlets were considered putative transgenic lines and used for subsequent molecular analyses. Putative transformants were first screened for T-DNA insertion by PCR using Cas9-specific primers (Table S2).

For mutation detection, a single primer pair was used to amplify a genomic fragment spanning the protospacer region and a diagnostic SNP that distinguishes the two alleles (Table S2). PCR products were first subjected to direct Sanger sequencing to screen edited lines. For genotype resolution, amplicons from selected lines were TA-cloned into the pMD19-T vector (TaKaRa, Dalian, China), and multiple independent colonies were sequenced. The diagnostic SNP within the amplicon was used to assign each clone to a specific allele, enabling unambiguous determination of allele-specific editing outcomes.

### 4.6. Salt Stress Treatment and Morphological Measurements

The *PdbRALF22-like* knockout line and wild-type poplar plants were grown on rooting medium supplemented with 0, 50, 100, or 125 mM NaCl for 1 month. Plant height, primary root length, and fresh weight were recorded at the end of the treatment, with three biological replicates per genotype per condition. For soil-based assays, one-month-old seedlings grown under non-saline conditions were transplanted into soil and acclimated in a greenhouse for an additional month. Plants were then irrigated with 200 mM NaCl for 5 days, while control plants received water. Morphological phenotypes of the mutant and wild-type plants were documented under control and salt stress conditions, with three biological replicates per genotype per treatment. At the completion of the treatment, shoot height was measured from the stem base to the shoot apex using ImageJ software (Version 1.54g, National Institutes of Health, USA). Primary root length was determined by measuring the distance from the root-shoot junction to the tip of the longest root. For fresh weight determination, whole plantlets were carefully harvested from the agar medium, gently rinsed with distilled water to remove residual media, blotted dry with filter paper, and immediately weighed on an analytical balance to the nearest 0.01 g.

### 4.7. Histochemical Staining

For the assessment of early salt-induced cellular damage, fully expanded detached leaves (the 3rd to 5th leaves from the apex) were collected from one-month-old soil-grown plants. These leaves were incubated in either a 0.2 M NaCl solution to induce salt stress or in distilled water as a control for 8 h prior to subsequent staining procedures.

Histochemical staining was performed to visualize reactive oxygen species accumulation and membrane damage in leaves. Hydrogen peroxide accumulation was detected by DAB staining, and superoxide accumulation was detected by NBT staining. Membrane damage and cell death were assessed by Evans Blue staining.

Staining solutions were prepared as follows: DAB, 1 mg mL^−1^ (3,3′-diaminobenzidine) adjusted to pH 3.8; NBT, 1 mg mL^−1^ (nitroblue tetrazolium) dissolved in 10 mM potassium phosphate buffer (pH 7.8); and Evans Blue, 0.25% (*w*/*v*) in ddH_2_O. Leaves from treated plants were immersed in the corresponding staining solutions and incubated on a horizontal shaker (100 rpm) at room temperature for 6 h in the dark. After staining, chlorophyll was removed by destaining in 95% ethanol overnight.

### 4.8. Evaluation of Fungal Pathogen Resistance and PdbPR1 Expression

Detached leaves from wild-type ‘Shanxin’ poplar and the *PdbRALF22-like* knockout line were inoculated with a conidial suspension of *Marssonina brunnea* f. sp. *monogermtubi* strain Ptom1 (5 × 10^5^ conidia/mL). A 30 μL droplet of the suspension was applied to the abaxial surface of each leaf. Inoculated leaves were placed in Petri dishes containing sterile filter paper moistened with ddH_2_O to maintain high humidity and incubated at 25 °C in the dark.

For fungal biomass quantification, genomic DNA was extracted from inoculated leaves. qPCR was performed using primers specific for the *M. brunnea* elongation factor 1-α gene (*MbEF1-α*) (Table S2), and the poplar elongation factor 1-α gene (*PdbEF1-α*) was used as an internal reference.

In parallel, under the same inoculation conditions, total RNA was extracted from leaf tissue and reverse-transcribed into cDNA. The expression of the defense marker gene *PdbPR1* was analyzed by RT-qPCR using gene-specific primers (Table S2), with *PdbEF1-α* as the reference gene.

Relative fungal biomass and gene expression levels were calculated using the 2^−ΔΔCT^ method. Statistical significance was determined by one-way ANOVA followed by Tukey’s post hoc test, based on three biological replicates.

### 4.9. Phylogenetic Analysis

FER homologs from poplar ‘Shanxin’ and *Populus trichocarpa* were identified by reciprocal BLASTP searches using *A. thaliana* FERONIA (AtFER) as the query. The resulting poplar candidates were included together with AtFER and functionally characterized FER proteins from other species, including apple MdFER [23] and *N. benthamiana* NbFER [24]. As outgroups, the *A. thaliana* CrRLK1L members ANXUR1 (AtANXUR1), ANXUR2 (AtANXUR2), and THESEUS1 (THE1) were included to root the tree (Table S3).

Protein sequences were aligned and the phylogeny was inferred using the maximum-likelihood method implemented in MEGA 7.0 [25] with the Jones–Taylor–Thornton (JTT) substitution model, using all sites and 1000 bootstrap replicates. A phylogenetic tree of PdbRALF family members identified in Section 2.2 was constructed using the same phylogenetic analysis method. Gaps and missing data of PdbFER family members were treated using the complete deletion option.

### 4.10. Statistical Analysis

All experiments were performed with at least three independent biological replicates. Specifically, the sample size (*n*) was 6 for the reactive oxygen species measurements and 3 for all other assays. Statistical analyses were conducted using SPSS software (Version 27.0). For comparisons among multiple groups, a one-way analysis of variance (ANOVA) followed by Tukey’s post hoc test was applied, whereas Student’s *t*-test was used for pairwise comparisons between two groups. Differences were considered statistically significant at *p* < 0.05.

## 5. Conclusions

In this study, we identified a RALF1/22/23/33-like subgroup in poplar ‘Shanxin’ and defined PdbRALF22-like as a unique member whose mature peptide rapidly elicits ROS in poplar leaf discs. Using a CRISPR/Cas9 knockout line, we demonstrate that loss of *PdbRALF22-like* enhances salinity tolerance but compromises resistance to the poplar leaf spot fungus, revealing a trade-off between abiotic and biotic stress outcomes. Transient assays further show that poplar FER-like receptors are sufficient to enable PdbRALF22-like-triggered ROS in *N. benthamiana*, supporting receptor-matched RALF–FER responsiveness. Together, our results establish PdbRALF22-like as a key node linking salt and immunity-related outputs in a woody species and provide a conceptual foundation for future precision engineering to fine-tune stress resilience in poplar under changing environments.

## Data Availability

The data supporting the findings of this study are provided in the main text and Supplementary Materials. Further inquiries can be directed to the corresponding author.

## Supplementary Materials

The following supporting information can be downloaded at: https://www.mdpi.com/article/10.3390/plants15101419/s1, Figure S1: Putative transformants were confirmed by PCR using Cas9-specific primers. Lane WT: Wild-type control; Lanes #1–#8: Putative transformants; Lane #9: Positive control (pYLCRISPR/Cas9-PdbRALF22-like binary vector); Table S1: List of PdbRALF-like; Table S2: Primers used in this study; Table S3: List of FERONIA homolog protein sequences, their loci and data sources used for constructing the PdbFER-like phylogenetic tree.
