# Supplementary material for: A RALF22-like Peptide Coordinates Salt Tolerance and Disease Susceptibility in Poplar (Populus davidiana × P. bolleana ‘Shanxin’)
Source: Plants (Basel). 2026 May 7;15(10):1419. doi: 10.3390/plants15101419 (PMC13211209; doi:10.3390/plants15101419)
Supplement: Supplementary file 1 [file plants-15-01419-s001.zip › Table S1.pdf]

Pdb RALF	Signal peptide	Coding sequence	protein	mature peptide
PdbRALF22-like	YES	ATGGCAAGGTGGAGTTCTTGGTTCTTGATTTCAGCTACAATCTTGATCTTGATGGCGATGGGCTTATCATCCACCGTTCAAGGAAGCGGGGATCACCATCTGGGGTGGATTCCTGCCACTAGATCATCAACCTGCAAGGGGTCTATAGCAGAGTGCATGGCTGAGGACGGAGAAGAGTTTGAGATGGACACAGAGATCAACCGGCGTATTTTAGCAACTAGCAAATACATCAGCTATGGTGCGCTTCAGAGGAACAATGTTCCTTGCTCTAGGCGTGGCGCTTCTTACTATAACTGCCAGAGGGGGGCTCAGGCTAATCCTTATAGCCGTGGATGCAGTAGCATTACAAGGTGCAGGAGTTGA	MARWSSWFLISATILILMAMGLSSTVQGSGDHHLGWIPATRSSTCKGSIAECMAEDGEEFEMDTEINRRILATSKYISYGALQRNNVPCSRRGASYYNCQRGAQANPYSRGCSSITRCRS	RRILATSKYISYGALQRNNVPCSRRGASYYNCQRGAQANPYSRGCSSITRCRS
PdbRALF1/23/33-like-1	YES	ATGACCACCTCAAAATATTACATTTTCCTTCTACTTTCTGCAATCCTAGCCGTCCATGTGTGCCTATCATCATCGACAGCAGTTGATTTTCTCCCCTTGGAGTCGTCGTCGGAGTGCCGGGGATCCATTGCTGAATGCTTGATGGATGACGAGTTTGGGATGGACACAGAGAGCAACAGGCGCATTCTAGCAACGTCGAGGTACGTAAGCTATGGTGCGCTGAGGAGGAACACTGTACCATGTTCGAGACGCGGCGCCTCGTATTATAATTGTCGACCTGGAGCTCAGGCCAATCCTTACTCTCGCGGGTGTAGTCGTATTACACGCTGCAGGAATTAA	MTTSKYYIFLLLSAILAVHVCLSSSTAVDFLPLESSSECRGSIAECLMDDEFGMDTESNRRILATSRYVSYGALRRNTVPCSRRGASYYNCRPGAQANPYSRGCSRITRCRN	RRILATSRYVSYGALRRNTVPCSRRGASYYNCRPGAQANPYSRGCSRITRCRN
PdbRALF1/23/33-like-2	NA	ATGGGAAACGATGAGTTTGAGAGGGACTGGGAAATCAACCGGCGGATATTGGCGACTAGCAACTACATAAGTTACGATGCTTTGGACAAGAACAACATACCATGTTCCCAGAGAGGTGCATCCTATTACAATTGCAAGACAGGTGCGCAGGCCAACCCTTATAGTCGTGGTTGCAGTGCTATTACTCGCTGCCGTAGTGGGTCCTTGCCTGCAATTTTCCTACCAGGAGCAGAGTATCTAATGAAAAGCATTAAGCTGCGAAGCAGAATAGATATAACAAGTGTTGTTCCAGCAAAACCTCCAACAGGTGAACACTTGAAAAGCCTTCCTAACTGGAAGCTCGTGCAAATACCAGAAATCCCATTTCAGGCAAATGAGGCAGTACGGATAGGCTCAGACTTGGTGGTTCGCCGTGATCAAAAGCTCATGTGCAGATAA	MGNDEFERDWEINRRILATSNYISYDALDKNNIPCSQRGASYYNCKTGAQANPYSRGCSAITRCRSGSLPAIFLPGAEYLMKSIKLRSRIDITSVVPAKPPTGEHLKSLPNWKLVQIPEIPFQANEAVRIGSDLVVRRDQKLMCR	RRILATSNYISYDALDKNNIPCSQRGASYYNCKTGAQANPYSRGCSAITRCRSG
PdbRALF1/23/33-like-3	YES	ATGGCGAGGCTGAGTTCTTTTTTCTTGATCTCCGCGACCGTCTTGATCTTGATGGTGATGGGCTTGCCATCCACCGTTCAAGGAAATGGGGACCACCACCACCACCTGGGGTGGATTCCGACCACCACAACCACCAGATCATCAATCTGCGACAAGGGGTCTTTAGCAGAGTGCATGGCTGAGGAGGACGGGGAAGAGTTTGGGATGGACACGGAGATCAACAGGCGCATTTTAGCAACTAGCAGTTACGTCAGCTACGGTGCGCTTCAGAAGAACAATGTTCCTTGCTCCAGGCGTGGTGCTTCTTACTATAATTGCAAGAATGGTGCTCAGGCTAATCCTTATAGTCGTGGATGCAGTCGCATTACAAGGTGCCGGGGTTGA	MARLSSFFLISATVLILMVMGLPSTVQGNGDHHHHLGWIPTTTTTRSSICDKGSLAECMAEEDGEEFGMDTEINRRILATSSYVSYGALQKNNVPCSRRGASYYNCKNGAQANPYSRGCSRITRCRG	RRILATSSYVSYGALQKNNVPCSRRGASYYNCKNGAQANPYSRGCSRITRCRG
PdbRALF-like-1	YES	ATGCCCATAATGGGTTCTAAGTTCTGGCTGGTTTTCCTCATGGTGGCTACGGCCTTGGTTGTTGAGTCGACAACTTTGATGGATGAAGCTGACCTCTGGGGCCTGTCCACTTTAGGCCATGACAGTAACGGTTTCGATTTCAGCACTGAGATGATGATGGATTCTGAGATCAATCACAGGTTGCTGGCTCAGAAGACAAGGTACATTAGCTATGGAGCCCTCAGGGCTAACTCGGTCCCTTGCAATCGTCGCGGTAGTTCTTACTACAACTGTAACAAGCGGCAAAAGGCTAATCCTTACAGACGTGGATGCAGCACCATCACAAGGTGCCGTCGGTACACTCATTAA	MPIMGSKFWLVFLMVATALVVESTTLMDEADLWGLSTLGHDSNGFDFSTEMMMDSEINHRLLAQKTRYISYGALRANSVPCNRRGSSYYNCNKRQKANPYRRGCSTITRCRRYTH	RLLAQKTRYISYGALRANSVPCNRRGSSYYNCNKRQKANPYRRGCSTITRCRRYTH
PdbRALF-like-2	YES	ATGCCCATAATGGGTTCTAAGTTCTGGCTGGTTTTCCTCATGGTGGCTACGGCCGTGGTTGTTGAGTCGACAACTTTGATGGATGAAGCTGAACTCTGGGGTCTGTCCACTTTAGGCCATGACAGTAACGGTTTCGATTTCAGCACTGAGATGATGATGGATTCTGAGATCAATCACAGGTTGCTGGCTCAGAAGACAAGGTACATTAGCTATGGAGCCCTGAGGGCTAACTCGGTCCCTTGCAATCGTCGCGGTAGTTCTTACTACAACTGTAACAAGCGGCAAAAGGCTAATCCTTACAGACGTGGATGCAGCACCATCACAAGGTGCCGTCGGTACACTCATTAA	MPIMGSKFWLVFLMVATAVVVESTTLMDEAELWGLSTLGHDSNGFDFSTEMMMDSEINHRLLAQKTRYISYGALRANSVPCNRRGSSYYNCNKRQKANPYRRGCSTITRCRRYTH	RLLAQKTRYISYGALRANSVPCNRRGSSYYNCNKRQKANPYRRGCSTITRCRR
PdbRALF-like-3	YES	ATGTCCAAGCTCAAATTTCTCCCTAACACCCCCCCATTCCATCTCTCACTGCTTTTCTTCCTCACCCTTTTCTCTGTTTGCAATGCTGATTCAGTGCGAGGCCTCAGTTCAATGAAAACCAGTGAGGTTGATGCCACCACTGTGAGTAGGGGTTGCTCCAATAAAATTGGAGAATGCTTTGAAGAGACAGAAATGGAGTCGGAGATCAGTAGGAGAGTGCTGCTGATGCAAAAGAGGTACATAAGTTACGGGACACTGAAGAGGGATATGGTTCCGTGTGATAAACCAGGAGCATCATACTATGATTGCAATGCTAGACAGGCTCATCCTTATAGCAGAGGTTGTGAGGTCATTACAAGGTGCGCAAGAAGCATCAATGACATTAACAATTAG	MSKLKFLPNTPPFHLSLLFFLTLFSVCNADSVRGLSSMKTSEVDATTVSRGCSNKIGECFEETEMESEISRRVLLMQKRYISYGTLKRDMVPCDKPGASYYDCNARQAHPYSRGCEVITRCARSINDINN	RRVLLMQKRYISYGTLKRDMVPCDKPGASYYDCNARQAHPYSRGCEVITRCAR
PdbRALF-like-4	YES	ATGTCCGAACTCCAGCTTTTTCCTCATACTCCCAAGCTACTCTACCTTTCACTGCTTTCCCTCCTCACCATTTTCACAATTTGCAATGCTGATTC	MSELQLFPHTPKLLYLSLLSLLTIFTICNADSVSGLNSLKTSEIDVTVSKGCSEKIGECFEEPE	RRVLLMQKKYISYETLRRDLVPCDKPG

Pdb RALF	Signal peptide	Coding sequence	protein	mature peptide
		AGTTTCAGGCCTCAATTCACTGAAAACCAGTGAGATTGATGTCACTGTCAGTAAGGGTTGCTCAGAGAAAATTGGAGAATGCTTTGAAGAGCC<br>AGAAATGGAGTCAGAGACCAGCAGGAGAGTGTTGCTGATGCAAAAGAAGTACATAAGTTATGAGACACTGAGGAGGGACTTGGTTCCCTGT<br>GATAAACCAGGAGCATCATACTATGACTGTAATGCTAGACAGGCTCATCCTTATAGCAGAGGCTGTGAGGTCATTACAAGGTGTGCAAGAAG<br>CGTCAAAGACATTAACAATTAG	MESETSRRVLLMQKKYISYETLRRDLVPCDKPGASYYDCNARQAHPYSRGCEVITRCARSV<br>KDINN	ASYYDCNARQAHPYSRGCEVITRCAR
PdbRALF-like-5	YES	ATGGCAGCTTCTACATTGCAATACCATCTCGCCTTCTTCATCTTCTTCCTCGTGATTGCTTCTTTCAGTCCAAGAATCCAAGCTCAAGTTGATG<br>AAACAAGCTTGAAGGCAATGAGAGATGCACTAGAATGGCCAATGTCCATGTATTATGATGAAGGCAGCGGTCTCGATGATGGGTTTGTGGGT<br>TTTGACGATGGGGCTGCTGAGGATGAAGAAAGTAGTCGTAGATCTTTGTTTTGGAGGAGAACGCGTTATTATATCTCGTATGGAGCTTTGTCT<br>GCTAATAGAATTCCTTGTCCAGCAAGGTCCGGGCGATCCTACTACAGCCACAATTGCTTCGCGTCTAGAGCTCCGGTGAATCCGTATTCCAG<br>AGGGTGTTCTAGGATCGCTCGTTGCAGGAGATGA	MAASTLQYHLAFFIFFLVIASFSPRIQAQVDETSLKAMRDALEWPMSMYYDEGSGLDDGFVG<br>FDDGAAEDEESSRRSLFWRRTRYYISYGALSANRIPCPARSGRSYYSHNCFASRAPVNPYS<br>RGCSRIARCRR	RRSLFWRRTRYYISYGALSANRIPCPA<br>RSGRSYYSHNCFASRAPVNPYSRGCS<br>RIARCRR
PdbRALF-like-6	YES	ATGGAACCAAAGTCCTTCCAATATTTTTGTTTCCTTCTAGTGATCTTCAGCCTTATAATAGCGTTGCTCAGCGACAGGGTTTCATCGAAAACTA<br>ATGAATGCAATGGTTCAATAGCAGAATGCAGTGAAGAGTATGAGTTCTTGATGCCATCTGATATCAGTAAAAGGTTTCTTGAAGAGAAGAGAA<br>AGTATATATCACCTGGTGCTTTGAAGCCAAACCGACCAGTTTGTAATGGTGGTGCTAGTGGTCAGTCATACAGTAGCAGTTGTCTTCCACCTC<br>CATCTAATCCTCCTTCTCGCGGTTGTTCCAAGTACTATCATTGTAGGTCAGATAATTGA	MEPKSFQYFCFLLVIFSLIIALLSDRVSSKTNECNGSIAECSEEYEFLMPSDISKRFLEEKRKYI<br>SPGALKPNRPVCNGGASGQSYSSSCLPPPSNPPSRGCSKYYHCRSDN	KRFLEEKRKYISPGALKPNRPVCNGGA<br>SGQSYSSSCLPPPSNPPSRGCSKYYH<br>CRS
PdbRALF-like-7	YES	ATGAGGCCTAGCGTAAGTATCGAATTCCTCCGGTGGCTCTCTTTAACTATCCTGCTGGTTTCTGTGATCACATCTGCTTCTACAGCTGCCTTT<br>CTTGAAAGCAACTCGAGTCCCATTTTCAATGCCACAATCGGTGAAGGTAATGAAGAGGAGTTCTCTATGGAATCTGAAGTGCATCAGTTCTCT<br>ATGGCCTCTCAGGGTAGCTATATTGGCTATAGGAGTTTAGAACGACAACCGATTTGCAATGCACAAATATATGGCGACTGTGCAAAACCGATC<br>AATCCTAACACTCGCCCTTGCACTTACTACAATCGGTGCAAACGTGGTAGTTGA	MRPSVSIEFLRWLSLTILLVSVITSASTAAFLESNSSPIFNATIGEGNEEEFSMESEVHQFSMA<br>SQGSYIGYRSLERQPICNAQIYGDCAKPINPNTRPCTYYNRCKRGS	FSMASQGSYIGYRSLERQPICNAQIYG<br>DCAKPINPNTRPCTYYNRCKRGS
PdbRALF-like-8	YES	ATGAGGGTTTCAGCTCTGAGTTTGATTGTTGCCTCCTTAACTTTTCTCATTGTTGTTAAAGCTCAGGTTGATTTGAAGGATTTCATCCAACTGA<br>CGAGTGAAGATCTTGAATGGCCATCAGCGTTGTCTGTCTACGATGAGTTAAGTGACAATGAAGACGGAGAGTCTGGCGGCGGGTCCCATGG<br>GAGGTCTCTGCATGGGAGAGCAAAGCACTATTACGTATCTTATGGAGCTCTCTCTGCAAACAGGGTGCCTTGCCCAGCTCGCTCAGGGAGG<br>TCTTACTATACCCATAACTGTTTCCGATCAAGAGGACAGGCTAACCCTTACACCAGAGGTTGCTCTTGTATCACTCACTGCAGGAGATAA	MRVSALSLIVASLTFLIVVKAQVDLKDFIQLTSEDLEWPSALSVYDELSDNEDGESGGGSHG<br>RSLHGRAKHYYVSYGALSANRVPCPARSGRSYYTHNCFRSRGQANPYTRGCSCITHCRR	HGRAKHYYVSYGALSANRVPCPARSG<br>RSYYTHNCFRSRGQANPYTRGCSCITH<br>CRR
PdbRALF-like-9	YES	ATGAGGCCTAGCGTAAGTATCGAATTCCTCCGGTGGCTCTCTTTAACTATCCTGCTGGTTTCTGTGATCACATCTGCTTCTACAGCTGCCTTT<br>CTTGAAAGCAACTCGAGTCCCATTTTCAATGCCACAATCGGTGAAGGTAATGAAGAGGAGTTCTCTATGGAATCTGAAGTGCATCAGTTCTCT<br>ATGGCCTCTCAGGGTAGCTATATTGGCTATAGGAGTTTAGAACGACAACCGATTTGCAATGCACAAATATATGGCGACTGTGCAAAACCGATC<br>AATCCTAACACTCGCCCTTGCACTTACTACAATCGGTGCAAACGTGGTAGTTGA	MRPSVSIEFLRWLSLTILLVSVITSASTAAFLESNSSPIFNATIGEGNEEEFSMESEVHQFSMA<br>SQGSYIGYRSLERQPICNAQIYGDCAKPINPNTRPCTYYNRCKRGS	VHQFSMASQGSYIGYRSLERQPICNAQ<br>IYGDCAKPINPNTRPCTYYNRCKRGS
PdbRALF-like-10	YES	ATGGGAAATTCTACATTGCAACACTATTTCACCTTCTTGATATTCCTCTTCACTATCTTTCTTGCTTTCAGTCCGAGAATCCAGGCGCAGGTCG<br>ATGAAACAAGCTTGAAGGCAATGGGCGATGCGCTAGAATGGCCGATGTCCATGTATTTCGATGAAAGTAGTGAGCTTGATGGTGGATTGATG<br>GATCTTGATGACGGTGAAGGAACTAGTCGTAGATCTTTGCTTTGGAGGAGAATGCATTACTACATCTCATATGGAGCTTTGTCTGCCAATAGA<br>ATTCCTTGCCCTGCAAGGTCCGGTAGATCTTACTATAGCCACAACTGCTTCAAGTCTAGGGTTCCTGTCAATCCCTACTCAAGAGGGTGTTCT<br>AGTATCACTCGTTGCCGGAGATGA	MGNSTLQHYFTFLIFLFTIFLAFSPRIQAQVDETSLKAMGDALEWPMSMYFDESSELDGGLM<br>DLDDGEGTSRRSLLWRRMHYYISYGALSANRIPCPARSGRSYYSHNCFKSRVPVNPYSRG<br>CSSITRCRR	RRSLLWRRMHYYISYGALSANRIPCPA<br>RSGRSYYSHNCFKSRVPVNPYSRGCS<br>SITRCRR
PdbRALF-like-11	YES	ATGGAACAGAAGTGCTTCCCTCATTTCTGTTTCCTCCTAGTGATCTTGAGCCTTGTAATATTGCAGCTCGGCGACAAGGTGGCGTCGAAAACG<br>AATGGATGCAATGGTTCGATAGCAGAATGCGATGAAGAATACGAGTTCTTGATGCCATCTCATGTTAGTAAAAGGTATCTTGAAGAGAAGAGG	MEQKCFPHFCFLLVILSLVILQLGDKVASKTNGCNGSIAECDEEYEFLMPSHVSKRYLEEKRK<br>YISPGALKPDQPVCNDGASGQSYSSSCLPPPSNSPSRGCSKYYRCRSDD	RYLEEKRKYISPGALKPDQPVCNDGAS<br>GQSYSSSCLPPPSNSPSRGCSKYYRC

Pdb RALF	Signal peptide	Coding sequence	protein	mature peptide
		AAGTATATATCACCTGGTGCTTTAAAGCCAGACCAGCCAGTTTGTAATGATGGCGCCAGTGGTCAGTCTTATAGTAGCAGTTGTCTTCCACCTCCATCTAATTCTCCTTCTCGCGGCTGTTCCAAGTACTATCGTTGCAGGTCTGATGATTGA		RS
PdbRALF-like-12	NA	ATGGAAACAACGAGGAACAGAAACATTTATTCCTTGCAGCTTTTTCTCGCAGCCTTCATATTAGTTCTAGTCCTTGTAGAACTGAGCTCCCAGTTTAGAGTTGCTGCTACGCAAGCAAGTACTGAACAAGTGCAATACAGTGGTTCCATGGTAGAATGTAGTGATGAAATGGCGGAGGAAGAATTATCAATGGAGTCAGAGACAAGTCGAAGAATTGTTAGAGCTGTCAAATATATTACTCCTGGTGTTTTGAGGTCCGACTCACCATTCTGTGGCAAGGTTAAGAGAGGCGAACCCTATCATGGCAGCTGCCTCCCTCCACCATCAAACAATTACAACAGAGGGTGCAACAAGTACTACAGGTGTAGGTCATGA	METTRNRNIYSLQLFLAAFILVLVLVELSSQFRVAATQASTEQVQYSGSMVECSDEMAEEELSMESETSRRIVRAVKYITPGVLRSDSPFCGKVKRGEPYHGSCLPPPSNNYNRGCNKYYRCRS	RRIVRAVKYITPGVLRSDSPFCGKVKRGEPYHGSCLPPPSNNYNRGCNKYYRCRS
PdbRALF-like-13	YES	ATGGAACCAAGGAGGAATATGTCTTATTCCTCGCAACGACTTCTTGCAGCTTTATTGATAATTCTAGGTCTCAGCTCCCAGTTTAGAGTTGCTGCTATGCAAGCAAGTTCTGAAAATGTGCATTGCAGTGGTTCTGTGGTAGAATTTAGTGGTCAGATGGCAGAAGGGGGATTATCAATGGAATCAGAGACAAGTCGAAGAACTGTTCGGGCTATCAAATTTATTACTCCTGGAGCTTTGAGGCCTGATGCACCATTCTGTGCCAAGGTTACAAGAGGTGAACCCTATAGTAATAATTGCCTACCTCCACCGTCAAACTCCTACAACAGAGGGTGCAATAACTACTACAGGTGCAGGTCATGA	MEPRRNMSYSSQRLLAALLIILGLSSQFRVAAMQASSENVHCSGSVVEFSGQMAEGGLSMESETSRRTVRAIKFITPGALRPDAPFCAKVTRGEPYSNNCLPPPSNSYNRGCNNYYRCRS	RRTVRAIKFITPGALRPDAPFCAKVTRGEPYSNNCLPPPSNSYNRGCNNYYRCRS
