# Supplementary material for: A RALF22-like Peptide Coordinates Salt Tolerance and Disease Susceptibility in Poplar (Populus davidiana × P. bolleana ‘Shanxin’)
Source: Plants (Basel). 2026 May 7;15(10):1419. doi: 10.3390/plants15101419 (PMC13211209; doi:10.3390/plants15101419)
Supplement: Supplementary file 1 [file plants-15-01419-s001.zip › Table S2.pdf]

Fragment name	Forward primer and reverse primers (5’ to 3’)	Note
PdbRALF22-like sgRNA	gtcaGATTAGCCTGAGCCCCCCTC<br>aaacGAGGGGGGCTCAGGCTAATC	Anneal and insert into BsaI sites of pYLsgRNA-AtU3d plasmid
PdbFER-like-1	ggggactctagaataggtaccATGGATAAGTGTTTCTGTGTTTCCG<br>gcccttgctcaccatctcgagTCATCTTCCTTTCGGGTTCATG	Amplify the *PdbFER-like-1* fragment
PdbFER-like-2	ggggactctagaataggtaccATGAGAAGCATGGATAAGTGTTTCTG<br>gcccttgctcaccatctcgagTCATCGTCCTTTCGGGTTCA	Amplify the *PdbFER-like-2* fragment
Cas9	GACAAGAAGTACTCCATCGGCC<br>CGAGGAGGTTGTCGAGATCA	Verify putative transformants
PdbRALF22-like	ATGGCAAGGTGGAGTTCTTGG<br>TCAACTCCTGCACCTTGTAATGC	Specifically amplify the *PdbRALF22-like* fragment to detect editing events
PdbPR1	TCAATGCCCACAATAATGCTCG<br>TAAGATCACCACTACCTCCTGC	Specific primers of ‘Shanxin’ *PdbPR1* for qPCR
PdbEF1-α	CCTGGACATCGTGACTTTATCA<br>GTCCATCTTGTTACAGCAGCAG	Internal control of ‘Shanxin’ for qPCR
MbMoEF1-α	TCACCGTGATTTCATCAAGAAC<br>GGCAACAGTCTTTGGGTTGTAT	Specific primers of *Marssonina brunnea* f. sp. *monogermtubi EF1-α*for qPCR
