# Supplementary material for: A RALF22-like Peptide Coordinates Salt Tolerance and Disease Susceptibility in Poplar (Populus davidiana × P. bolleana ‘Shanxin’)
Source: Plants (Basel). 2026 May 7;15(10):1419. doi: 10.3390/plants15101419 (PMC13211209; doi:10.3390/plants15101419)
Supplement: Supplementary file 1 [file plants-15-01419-s001.zip › Table S3.pdf]

Name	Locus	Data availability
AtANXUR1	AT3G04690	Tair
AtANXUR2	AT5G28680	Tair
AtFER	AT3G51550	Tair
AtTHESEUS1	AT5G54380	Tair
MdFER	XP_028957174.1	NCBI
NbFER	Niben101Scf07619g00006.1	https://solgenomics.net
PdbFER-like-1	JANKYH010000005.1	NCBI
PdbFER-like-2	JANKYH010000327.1	NCBI
PtriFER-like-1	XP_002308259.2	NCBI
PtriFER-like-2	XP_002323088.2	NCBI
