# Supplementary figures and images for: A RALF22-like Peptide Coordinates Salt Tolerance and Disease Susceptibility in Poplar (Populus davidiana × P. bolleana ‘Shanxin’)
Source: Plants (Basel). 2026 May 7;15(10):1419. doi: 10.3390/plants15101419 (PMC13211209; doi:10.3390/plants15101419)

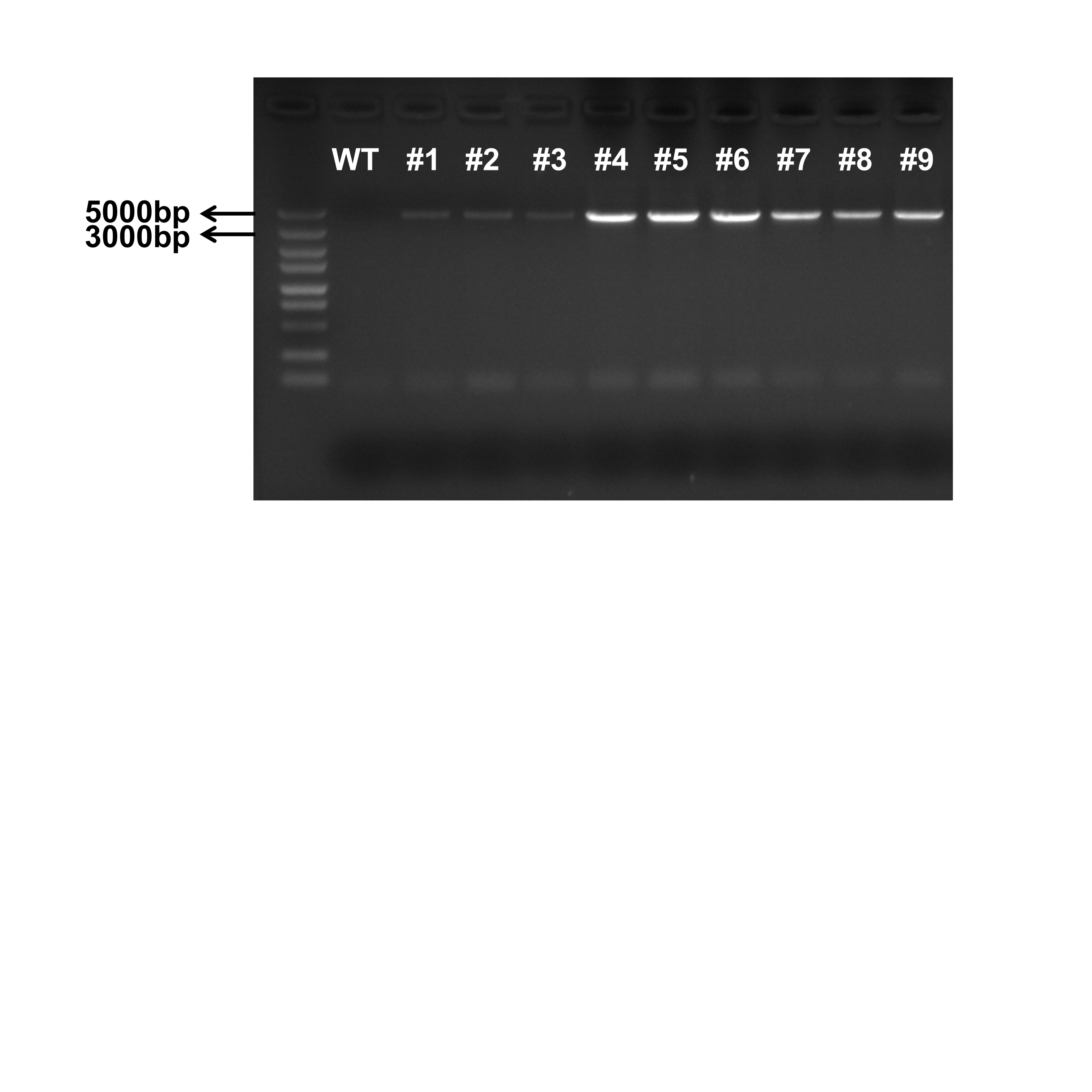

Supplement: Supplementary file 1 [file plants-15-01419-s001.zip › Figure S1.tif]
